# Whole-Genome Selective Scans Detect Genes Associated with Cashmere Traits and Climatic Adaptation in Cashmere Goats (*Capra hircus*) in China

**DOI:** 10.3390/genes16030292

**Published:** 2025-02-27

**Authors:** Hongying Dan, Hai’an Zhong, Zhanerke Akhatayeva, Kejian Lin, Songsong Xu

**Affiliations:** 1 Frontiers Science Center for Molecular Design Breeding (MOE), State Key Laboratory of Animal Biotech Breeding, College of Animal Science and Technology, China Agricultural University, Beijing 100193, China; dandandan@cau.edu.cn (H.D.); hazhong@cau.edu.cn (H.Z.); 2 Institute of Grassland Research of Chinese Academy of Agricultural Sciences, Hohhot 010010, China; akhatayevazhanerke@163.com

**Keywords:** goats, cashmere traits, climatic adaptation, genetic markers

## Abstract

**Background:** Cashmere, valued for its exceptional softness and warmth, is a major focus in goat breeding due to its high economic importance. However, the molecular mechanisms underlying cashmere production remain largely unknown, hindering efforts to optimize yield and quality. Additionally, domestic goats exhibit remarkable adaptability to diverse climates, ranging from arid northern regions to humid southern areas, yet the genetic basis for these adaptations is poorly understood. Exploring the genetic factors driving cashmere production and climatic adaptation could provide crucial insights into trait evolution and support the development of breeding strategies for improved productivity and resilience. **Methods**: We utilized whole-genome resequencing data from 157 samples representing 14 goat populations to analyze the genetic diversity between cashmere and non-cashmere breeds. Additionally, we conducted the tests of selective sweeps (i.e., pairwise *F_ST_*, *θ*π and XP-EHH) for cashmere traits and genome–environment association analysis (i.e., XtX statistic), respectively. **Results**: We identified strong selective signatures in previous reports (e.g., *AKT3*, *FOXP1*, *FGF5*, *TGFBR3*) and novel genes (e.g., *ZEB1*, *ZNRF3*, *MAPK8IP3*, *MAPK8IP2*, *AXIN1*) associated with cashmere traits. Further gene annotation and KEGG analyses showed that these genes were identified to be the most probable genes accounting for the cashmere traits. Also, we detected some genes such as *PDGFRB*, *PRDM8*, *SLC26A2*, *SCAMP1*, *EPHX1*, *CDC25A*, and *POLK* that played critical roles in the adaptation of goats to local climate variation. **Conclusions**: Collectively, our results provide novel insights into the genetic mechanisms underlying the cashmere traits and climatic adaptation, and also identified new genetic markers for genetic improvement in goats.

## 1. Introduction

The domestic goat *(Capra hircus*) is thought to have originated from a mosaic of wild bezoar populations (*Capra aegagrus*) as early as 10,500 years ago in the region around the Zagros Mountains, which is now part of western Iran [1]. Following domestication, goats quickly spread from their center of origin to various parts of the world [2]. Today, more than 300 distinct goat breeds exist globally, exhibiting diverse morphological and production traits, such as variations in cashmere quality. These characteristics have been shaped by factors including human selection, genetic isolation, the founder effect, and genetic drift [3,4].

Among these breeds, cashmere goats are particularly notable for their dual hair follicle types: primary follicles produce coarse outer hair, while secondary follicles generate fine cashmere fibers. These fibers, prized as luxury materials, are valued for their exceptional fineness, softness, lightness, and superior insulation compared to sheep’s wool. Previous research has uncovered various genes and genetic variants linked to cashmere quality and yield, primarily using low-density Single Nuncleotide Polymorphism (SNP) arrays or whole-genome sequencing approaches. For example, Qiao et al. conducted a genome-wide association study (GWAS) on cashmere fineness in Inner Mongolian Cashmere goats (Erlangshan type) using a 66K SNP capture chip, identifying four significant loci within the genes *AKT1*, *ALX4*, *HK1*, and *NT-3* [5]. This chip is the first domestically developed chip that can be used for genome and genetic diversity analysis in cashmere goats. Similarly, Cai et al. employed whole-genome sequencing to analyze ancient genomes, shedding light on the evolutionary history of Chinese cashmere goats [6]. Han et al. identified a 582 bp deletion 367 kb upstream of LHX2 through whole-genome analysis, which may be linked to cashmere yield and fiber diameter [7]. In addition, genetic diversity and adaptation characteristics vary significantly among cashmere goat populations in different regions. For instance, Changthangi goats, renowned for producing some of the finest cashmere globally, have shown high genetic diversity (observed heterozygosity of 0.75) and low population differentiation (*F_ST_* value of 0.0531) based on 15 microsatellite markers, indicating genetic stability within the population [8]. Similarly, studies on Mongolian cashmere goats revealed substantial phenotypic diversity and low genetic differentiation among breeds (*F_ST_* value of 0.017). Genome-wide association studies (GWAS) in these populations have identified significant genes associated with traits such as body weight, cashmere quality, and coat color [9]. These findings provide valuable insights into genetic improvement strategies but fall short of systematically comparing the genetic structure across different cashmere goat breeds in China.

Adaptation to local environments is another essential trait of cashmere goats, which are typically found in colder regions. In China, for instance, cashmere goats are primarily distributed in the colder northern areas. This ability to thrive in harsh, chilly environments highlights the significance of their physiological and genetic adaptations, enabling them to endure extreme climates while producing high-quality cashmere. Adaptation is one of the most striking features of the biological world, and is an essential capability of an organism to survive in diverse environments. Indigenous goats have undergone extensive adaptation to local conditions, including extreme environments, over hundreds or even thousands of years, achieving a homeostasis finely tuned to their ecological demands [10,11,12,13]. For instance, cashmere goats raised in northern China exhibit remarkably dense hair coats and a more compact body structure compared to their counterparts in southern China [14,15]. And the adaptation of Tibetan cashmere goats to high-altitude, low-oxygen environments and the metabolic complexities affect variations in cashmere fiber diameter [16]. Advances in sequencing and genotyping technologies have facilitated the identification of genomic changes linked to goat adaptation to diverse environmental conditions. Key genes have been implicated in high-altitude adaptation [17,18], responses to hot climates and immune challenges [19], as well as lipid metabolism, hypoxia stress, lung function, seasonal behaviors, and neuronal function [20]. For instance, the endothelial PAS domain protein 1 (*EPAS1*) regulates the hypoxia-inducible factor 1 (*HIF-1*) transcriptional complex, promoting angiogenesis, erythropoiesis, and metabolic shifts to enhance oxygen delivery to skeletal muscles [21]. Similarly, TNF (Tumor necrosis factor) receptor-associated protein 1 (*TRAP1*) contributes to mitochondrial metabolism by inducing *HIF1α* transcription and preventing its proteasomal degradation [22]. Nevertheless, how goats can thrive in a wide range of varying climatic and environmental regions remains poorly understood.

The purpose of this experiment was to explore the genetic basis of cashmere traits and local adaptation in Chinese cashmere goats. To achieve this, we collected a total of 157 samples of 14 domestic goat breeds and climatic data which was composed of the observations of 6 climatic variables over 30 years. We characterized the patterns of genetic variations in terms of between-population genetic relationships and within-population genetic diversity. To uncover the genetic basis of cashmere traits, we conducted genome-wide selective scans to identify key associated genes. Furthermore, genome–environment association analyses were performed to detect climate-related genetic variations by integrating molecular and climatic datasets. Our findings provide valuable insights into local adaptation mechanisms and the genetic architecture underlying cashmere traits in goats.

## 2. Materials and Methods

### 2.1. Genotyping and Quality Control

**Figure 1:**
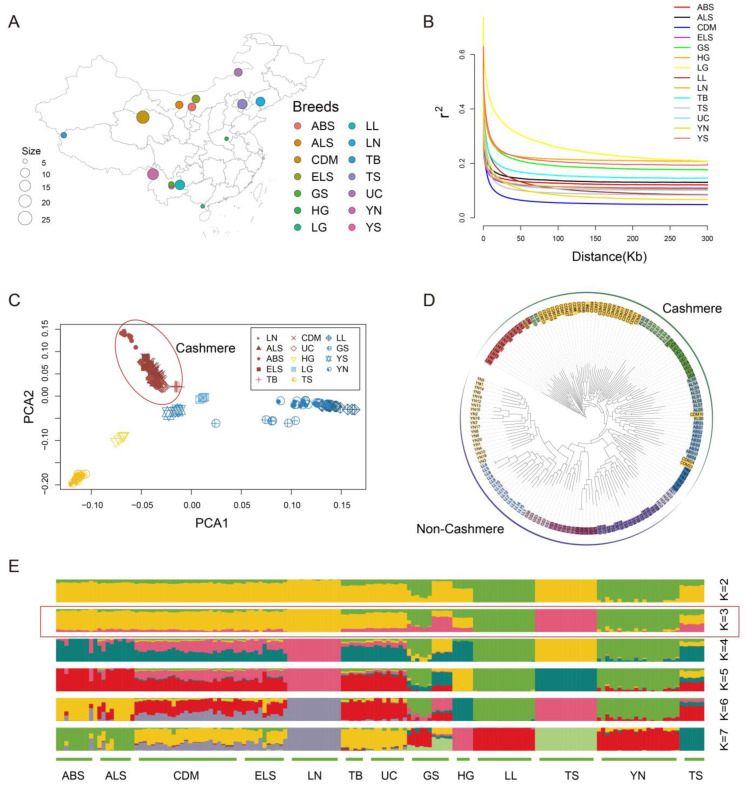
Population relationship and structure of cashmere goats and non-cashmere goats. (**A**) Geographical distribution of domestic goat breeds in China. (**B**) LD decay analysis. (**C**) Principle component analysis of 157 goats. (**D**) NJ tree constructed by identity-by-state matrix among 157 samples. (**E**) Population structure of 14 goat breeds revealed by ADMIXTURE analysis (K  =  2 to 7), K = 3 is optimal value (red reframe).

We collected 157 individuals from 14 domestic goat populations, representing 7 cashmere goat breeds: Liaoning Cashmere goat (LN), Arbus cashmere goat (ABS), Erlangshan cashmere goat (ELS), Alashan cashmere goat (ALS), Tibetan cashmere goat (TB), Chaidamu cashmere goat (CDM), and Ujumqin cashmere goat (UC) and 7 non-cashmere goat breeds: Huai goat (HG), Leizhou goat (LG), Tangshan dairy goat (TS), Longlin goat (LL), Yunshang black goat (YS), Yunnan black bone goat (YN), and Guishan goat (GS). This study aimed to investigate genetic variants shaped by long-term natural and artificial selection (Figure 1A, Supplementary Table S1). Whole-genome resequencing data for these individuals were obtained from the publicly available NCBI database (National Center for Biotechnology Information, https://www.ncbi.nlm.nih.gov/, accessed on 15 July 2024).

We filtered the raw reads using three criteria: (1) reads with unidentified nucleotides (N) more than 10%; (2) reads containing adapter and poly-N; (3) reads whose low-quality base ratio is more than 50%. The clean reads were aligned to the goat reference genome ARS1.2 (GCA_001704415.1) using the Burrows–Wheeler Aligner (BWA) v0.7.17 MEM module [23], with the parameters “bwa -k 32 -M -R”. SAMtools v1.17 was used to convert the file format from SAM to BAM and to filter out unmapped and non-unique reads [24]. Following mapping, SNP calling was performed following the GATK Best Practices workflow with a joint genotyping approach [25]. The process consisted of two steps: (1) variant calling for each sample using the HaplotypeCaller module with the parameters --genotyping-mode DISCOVERY --min-base-quality-score 20 --output-mode EMIT_ALL_SITES --emit-ref-confidence GVCF; and (2) joint genotyping, where all GVCFs were combined using the GenotypeGVCFs and CombineGVCFs modules. Variant sites were filtered based on the following criteria: QUAL < 30.0, QD < 2.0, MQ < 40.0, FS > 60.0, HaplotypeScore > 13.0, MQRankSum < −12.5, and ReadPosRankSum < −8.0, using the VariantFiltration module in GATK. SNPs were called separately for each species and then merged by Combine GVCFs and jonintly genetyped by GenotypeGVCFs in GATK. Strict quality control of the SNP dataset was applied using PLINK v1.09 [26], and individuals and SNPs were excluded if they met any of the following conditions: (i) SNPs without chromosomal or physical positions; (ii) SNPs with >10% missing data; (iii) individuals with genotyping rates < 90%; (iv) minor allele frequency (MAF) < 0.05; and (v) *p*-value for Hardy–Weinberg equilibrium (HWE) < 0.001.

### 2.2. Genetic Diversity and Population Genetic Structure

We assessed the genomic diversity for the populations of goat based on four metrics including observed heterozygosity (*H_o_*), expected heterozygosity (*H_e_*), inbreeding coefficient (*F*_ROH_), with calculations performed in PLINK v1.09 [26]. We investigated the levels of linkage disequilibrium (LD) decay between pairs of autosomal SNPs with the *r*^2^ estimate using PopLDdecay v3.41 software [27].

To examine population genetic structure, principal component analysis (PCA) was conducted using PLINK v1.90 to explore genetic relationships among breeds. The PCA results were visualized using the plot function in R v4.4.0. A neighbor-joining (N-J) tree was generated using FastTree 2.1 and Tassel 5.0 [28], and the tree topology was visualized in iTOL [29]. The robustness of the tree topology was assessed by performing 1000 bootstrap replicates. Lastly, the genetic structure of the populations was further explored using the maximum-likelihood clustering program ADMIXTURE v1.30, with K values ranging from 2 to 7 [30].

### 2.3. Genomic Selection Signals Analysis

To identify the genomic signatures of selection between cashmere goats and non-cashmere goats of the cashmere trait, we calculated the genetic differentiation (*F_ST_*) values and nucleotide diversity scores (*θ*π) for the 50 kb sliding window and 100 kb intervals along the chromosomes using Vcftools v0.1.17. Furthermore, we calculated the the cross-population extended haplotype homozygosity (XP-EHH) scores for the 50 bp intervals along the chromosomes using selscan v1.3.0. For each chromosome, XP-EHH scores were averaged per window across non-overlapping 50 kb windows. The top 5% of values from all three methods were selected as candidate outliers under strong selective sweeps.

### 2.4. Signatures for Local Climatic Adaptation

To investigate local adaptation signatures in cashmere goats, we performed a genome–environment association analysis. The correlations between SNPs and climatic variables were calculated using the XtX statistic in BayPass v2.2, which integrates population genetics, ecological modeling, and statistical learning techniques [31]. To establish a calibrated threshold (0.01%), we simulated pseudo-observed datasets with 5,000,000 SNPs, allowing us to identify SNPs potentially under selection, referred to as adaptive SNPs.

For identifying environmental associations of the adaptive SNPs, we selected six climate variables: annual mean temperature, maximum temperature of the warmest month, minimum temperature of the coldest month, total annual precipitation, precipitation of the driest month, and annual precipitation (Table 1, Supplementary Table S2). These climatic data, spanning from 1970 to 2000, were obtained from WorldClim (https://www.worldclim.org/data/worldclim21.html#, accessed on 10 July 2024) at a spatial resolution of 1 km for the specific coordinates of each population. Climate variables were extracted using ArcGIS v10.4.1 software, which enabled high-resolution visualization of the annual mean climate variations at the sampling sites.

### 2.5. Functional Enrichment Analyses

The 50 kb upstream and downstream regions of significant SNP loci were defined as candidate regions under selection based on the goat reference genome annotation. Gene ontology (GO) enrichment and KEGG pathway analyses were performed for the annotated genes, using the goat genome as the background in the DAVID Bioinformatics Resources v.6.8 [32]. A threshold of Q < 0.05 was applied, and categories with at least two genes from the input list were considered significantly enriched in GO terms and KEGG pathways. To visualize the results, we generated word clouds for the enriched GO terms and KEGG pathways using the Wordcloud generator (https://www.jasondavies.com/wordcloud/, accessed on 20 September 2024).

## 3. Result

### 3.1. Genome-Wide SNPs’ Detection and Annotation

After stringent data quality control, a total of 12,716,092 high-quality autosomal SNPs were retained for downstream annotation and analysis. Resequencing data yielded an average depth of 13.11× across the genome, ensuring reliable variant detection. Furthermore, 99.84% of the reads were successfully mapped to the latest goat reference genome, ARS1.2 (GCA_001704415.1), confirming high alignment accuracy (Supplementary Table S1).

The SnpEff program [33] was utilized to annotate high-quality SNPs based on the ARS1.2 reference genome. SNPs were categorized according to their genomic locations, including exonic, intronic, intergenic, upstream, downstream, and splicing regions. Among these, transcript regions constituted the largest portion of SNPs, accounting for 34.46% of the total data, closely followed by intronic regions, which made up 33.88%. Exonic regions represented only 0.58% of the dataset, encompassing 110,187 synonymous mutations. These classifications provide insight into the functional impact of SNPs within various genomic regions, which may contribute to phenotypic diversity in the studied goat populations.

### 3.2. Genomic Diversity and Structure of Domestic Goat Population

To further evaluate the genetic diversity of cashmere goats in comparison to other Chinese goat breeds, we calculated the *H_e_*, *H_o_*, and *F*_ROH_ for each breed. Analysis of *H_o_* and *H_e_* revealed that the *H_o_* in cashmere goat breeds—specifically ABS, ALS, CDM, ELS, LN, and TB—was lower than their *H_e_* suggesting potential inbreeding within these groups (Table 2). Notably, the LN breed exhibited the highest inbreeding coefficient (*F*_ROH_ = 0.541), followed by ALS (*F*_ROH_ = 0.523), while the GS breed displayed the lowest inbreeding level (*F*_ROH_ = 0.036).

In the linkage disequilibrium (LD) decay analysis, the slowest LD decay was observed in LG, followed by HG and YS, while CDM exhibited the fastest decay (Figure 1B). Generally, slower LD decay rates are associated with lower domestication levels and stronger natural selection pressures. These findings suggest that CDM has undergone artificial breeding with lower selection intensity compared to other breeds, contributing to its higher genetic diversity.

PCA revealed a distinct geographic clustering pattern among domestic goat populations (Figure 1C). Cashmere goat populations and non-cashmere goat populations separated clearly. In the cashmere goat populations, Inner Mongolia cashmere goats (ALS, ABS, ELS) and Chaidamu cashmere goats were close to each other. In contrast, non-cashmere goat populations showed a clear division: northern populations (HG, TS) were distinct from southern populations (YN, YS, GS, LL, LG). The NJ tree reinforced the PCA results, showing a clear clustering of cashmere goats with closer genetic relationships (Figure 1D). ADMIXTURE analysis (Figure 1E) with K = 3 (optimal CV), within the cashmere goat populations, Inner Mongolia cashmere goats (ALS, ABS, ELS) clustered closely with each other. Except for the LN breed, other cashmere goat populations showed genetic admixture, likely reflecting crossbreeding between Liaoning cashmere goats and local breeds in their breeding history. Overall, cashmere goat breeds in China have unique genetic structure.

### 3.3. Genome-Wide Selective Sweeps

**Figure 2:**
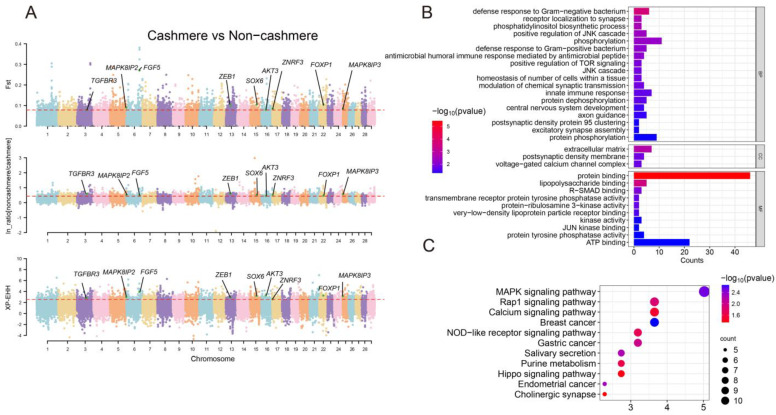
Genomic selection sweep signals in domestic goats. (**A**) Manhattan plot illustrating the genome-wide distribution of selection metrics, including *F_ST_*, ln-ratio *θ*π, and XP-EHH, between cashmere and non-cashmere goats. The dotted red line representing the top 5% cutoff for each metric. (**B**) Gene ontology (GO) terms for genes under selection in cashmere goats. (**C**) KEGG pathways enrichment analysis for selected genes in cashmere goats.

In order to identify significant selectives linked to cashmere traits, we compared the genetic differentiation between cashmere and non-cashmere goat populations. This was achieved by estimating *F_ST_*, ln-ratio *θ*π, and XP-EHH along the genome. Through this analysis, we identified 2357, 3055, and 2045 genes by estimating *F_ST_*, ln-ratio *θ*π, and XP-EHH, respectively (Figure 2A, Supplementary Tables S3–S5). Notably, we found significant signals associated with both previously known and newly identified functional genes. A total of 322 genes were shared between the three selection scan metrics (Supplementary Table S6). For example, Gong et al. [34] reported that *AKT3* expression was significantly lower in long-haired Inner Mongolian cashmere goats and suggested that it might suppress hair growth. Comparative study identified the *FGF5* genes as a candidate that might improve goat fiber traits [35]. Jin et al. [36] revealed the *forkhead box P1* (*FOXP1*) gene in cashmere goats that is crucial for preserving quiescence of hair follicle progenitor cells. These genes were also found in our study. We specifically identified novel genes related to cashmere traits in goats, including *ZEB1*, *ZNRF3*, *MAPK8IP3*, *MAPK8IP2*, and *AXIN1*.

There are 31 significantly enriched (*p* < 0.05) GO terms associated with selected genes, involving defense response to Gram-negative bacterium, receptor localization to synapse, phosphatidylinositol biosynthetic process, positive regulation of JNK cascade, phosphorylation, defense response to Gram-positive bacterium, antimicrobial humoral immune response mediated by antimicrobial peptide, positive regulation of TOR signaling, and JNK cascade (Figure 2B, Supplementary Table S7). KEGG enrichment analysis revealed that these genes are strongly linked to several biologically significant pathways, including breast cancer, MAPK signaling pathway, endometrial cancer, Salivary secretion, Gastric cancer, Rap1 signaling pathway, NOD-like receptor signaling pathway, purine metabolism, calcium signaling pathway, Hippo signaling pathway, and cholinergic synapse (Figure 2C, Supplementary Table S8).

### 3.4. Genome-Wide Scans for Local Climate Adaptation

**Figure 3:**
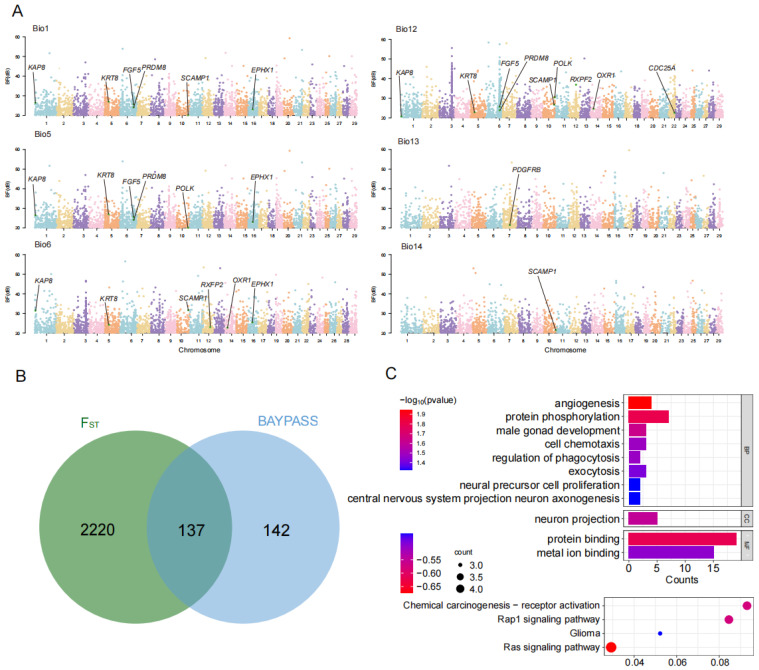
Genetic signatures associated with local climatic adaptation in domestic goats. (**A**) Manhattan plot displaying the results of BayPass analysis, highlighting loci with strong evidence of selection (Bayes Factor, BF > 20) potentially associated with adaptation to local climatic variables. (**B**) Venn diagram comparing selected genes identified through *F_ST_* and BayPass analyses. (**C**) GO terms and KEGG pathways enrichment analysis for selected genes in cashmere goats.

To identify genes associated with local climate adaptation, we focused on Chinese cashmere goats and conducted genome–environment association (GEA) analyses using BayPass [31]. Genes containing at least one SNP with a BF > 20 (which indicates decisive evidence for association according to Jeffreys’ rule [37]) with a specific environmental covariate were considered potential candidate genes (Figure 3A). BayPass identified 1079 SNPs putatively under selection for six climate variations and found 279 functional genes located at or near (within 50 kb) these SNPs. These genes were presumably involved in cancer (*SMG6*, *HCK*), cellular growth (*CPVL*), reproduction (*ADGB*, *CRISP1*, *CTTNBP2NL*, *WDR78*), and nervous system (*AUTS2*, *CNTNAP2*, *CPVL*, *SRGAP2*).

Building on those insights, we further identified 137 specific genes under selection by *F_ST_* and BayPass (Figure 3B, Supplementary Table S9). We identified genes associated with cashmere traits, such as *KAP8*, *KRT8*, *FGF5*, and *PDGFRB*. Additionally, we found that genes *PRDM8*, *SLC26A2*, *SCAMP1*, *EPHX1*, *CDC25A*, *POLK*, *RXFP2*, and *OXR1* are involved in adaptation to local climates. For example, *RXFP2* and *OXR1* had been reported to be involved in heat and cold adaptation [13,38]. The downregulation of PRDM8 is proposed as a potential mechanism of transcriptional regulation, akin to the previously reported effect of hypoxia-inducible factor 1α in high-altitude environments [39]. *SCAMP1* was found to be associated with the hypoxia response and ultraviolet radiation damage [40].

GO enrichment analysis was conducted on the 137 candidate genes associated with environmental factors to assess their functional enrichment across various biological processes, molecular functions, and KEGG pathways. We revealed 11 GO terms using a threshold of *p* < 0.05 (Figure 3C, Supplementary Table S10). In KEGG enrichment analyses, these genes were found to be closely associated with several biologically important pathways (*p* < 0.05), melanoma, Ras signaling pathway, glioma, Rap1 signaling pathway, and chemical carcinogenesis—receptor activation (Supplementary Table S10).

## 4. Discussion

In this study, we used resequenced data of the genomes of 85 cashmere goats and 72 non-cashmere goats. Population genetic analysis revealed a clear separation between cashmere and non-cashmere goat populations. However, Inner Mongolia cashmere goats (IMC) and Chaidamu cashmere (CDMC) goats were close to each other. This finding aligns with Han et al. [7], who reported that IMC and CDMC belong to the northern cashmere goat populations, distinct from other ordinary goat populations. The mixed phenomena of Liaoning flocks with others due to the fact that most of the cashmere goat breeds in China were obtained by crossbreeding Liaoning goats. For instance, Shaanbei White cashmere goats were obtained by crossing Liaoning cashmere and Shaanbei Ziwuling Black goats [41].

A genome-wide selection scan analysis demonstrated selective sweep regions in cashmere goats containing genes such as *AKT3*, *MUC6*, *FGF5*, *FOXP1*, *ZEB1*, *TGFBR3*, *ZNRF3*, *MAPK8IP3*, *MAPK8IP2*, and *AXIN1.* Gong et al. [34] reported that *AKT3* expression was significantly lower in long-haired Inner Mongolian cashmere goats and suggested that it might suppress hair growth. The PI3K-Akt signaling pathway has been implicated in the de novo renewal of hair follicles [42]. *ZEB1*, a zinc-finger transcription factor, regulates melanocyte differentiation through the WNT, TGF-β, and Notch signaling pathways [43], suggesting it may influence cashmere fiber coloration. Transforming growth factor receptor β 3 (*TGFBR3*), a key component of the TGF-β signaling pathway, may be involved in hair follicle development [44]. Zinc and ring finger 3 (*ZNRF3*) has been shown to regulate Wnt signaling [45], a pathway crucial for hair follicle morphogenesis [44]. Additionally, *AXIN1*, which plays a central role in integrating WNT/β-catenin signaling, Hippo signaling, and TGFβ signaling, could also contribute to the mechanisms of cashmere production [46]. Mitogen-activated protein kinase 8 interacting protein 3 (*MAPK8IP3*) and mitogen-activated protein kinase 8 interacting protein 2 (*MAPK8IP2*) are the members of the MAPK signaling, which is required for hair follicle growth phase and stem cell inactivity [47]. Interestingly, our study identified the mucin 6 (*MUC6*) gene, which was associated with domestication origin in goat [48]. These findings suggest that cashmere traits are likely influenced by genes involved in hair follicle development and provide theoretical support for further functional experiments [49].

Through genome-wide scans for local adaptation, we identified 137 candidate genes that were significantly associated with climate factors. Among these, genes such as *KAP8*, *KRT8*, *FGF5*, and *PDGFRB* have been reported to be associated with the production mechanisms of cashmere [50,51,52]. Northern China experiences a relatively cold and dry climate, while southern China is characterized by a hotter and more humid climate [53]. Northern goats, particularly those bred for cashmere production, have dense coats and more compact bodies compared to their southern counterparts [54]. It indicated that the environment may have an influence on the growth of cashmere. Several genes related to hypoxia response, including *PRDM8*, *SLC26A2*, *SCAMP1*, and *EPHX1* were also identified [39,40,49,55]. Notably, *EPHX1* plays a key role in regulating hypoxia-induced genes that affect blood pressure and circulation [55]. *SCAMP1*, *CDC25A*, and *POLK* are associated with responses to ultraviolet (UV) radiation. *SCAMP1* is linked to UV damage resilience [40], while *CDC25A* deletion promotes apoptosis and enhances DNA damage repair following UV exposure [56]. *POLK*, a DNA polymerase involved in nucleotide excision repair and trans-lesion synthesis, plays a crucial role in repairing ultraviolet-induced DNA damage. Its ability to localize to damage sites is dependent on the catalytic activity of METTL3 [57]. Additionally, *FBXL21* was detected to be linked to mammalian circadian rhythms in sheep [58]. These findings suggest that these genes may contribute to local adaptation in Chinese goats, supporting the differentiation between northern and southern goat populations and potentially influencing cashmere development.

## 5. Conclusions

Our study identified several key genes likely influencing both cashmere yield and quality, as well as the local environmental adaptation of goats. Genes such as *AKT3*, *FOXP1*, *FGF5*, *TGFBR3*, *ZEB1*, *ZNRF3*, *MAPK8IP3*, *MAPK8IP2,* and *AXIN1* appear crucial for hair follicle development and cellular signaling, impacting the fiber traits critical for cashmere production. Additionally, genes like *KAP8*, *KRT8*, *FGF5*, and *PDGFRB* along with *PRDM8*, *SLC26A2*, *SCAMP1*, *EPHX1*, *CDC25A*, *POLK*, *RXFP2*, and *OXR1* are associated with responses to environmental stresses, including UV radiation repair, hypoxia, and oxidative resilience. These genes may contribute to cashmere goats’ adaptation to regional climates, indicating genetic foundations for traits that support survival and performance in diverse environments.

While further experimental validation is needed to clarify the specific mechanisms, these findings lay a valuable foundation for advancing cashmere goat breeding. Our study highlights actionable genetic targets for enhancing both cashmere quality and adaptability, with significant potential for supporting sustainable growth in the Chinese cashmere industry.

## Figures and Tables

**Table 1:** Six climate variables of each population.

Population	Bio1	Bio5	Bio6	Bio12	Bio13	Bio14
LN	8.6	26.9	−13.9	679.0	78.0	2.0
LL	18.5	28.5	5.8	1213.0	62.0	2.0
TS	11.5	29.7	−11.1	615.0	189.0	6.0
YN	8.7	17.9	−4.6	948.0	201.0	3.0
YS	15.1	24.0	2.4	976.0	33.0	2.0
CDM	4.2	24.1	−18.5	142.0	75.0	2.0
UC	1.7	27.1	−26.2	301.0	31.0	1.0
ELS	7.2	30.2	−17.6	223.0	13.0	0.0
ABS	7.8	29.7	−16.6	259.0	198.0	13.0
ALS	9.0	31.3	−15.5	129.0	158.0	15.0
HG	15.1	31.7	−3.2	848.0	233.0	14.0
LG	24.0	32.5	13.9	1676.0	192.0	13.0
TB	−1.5	18.9	−21.9	32.0	188.0	14.0
GS	14.0	23.3	0.9	996.0	285.0	21.0

**Table 2:** The expected heterozygosity (*H_e_*), observed heterozygosity (*H_o_*), and inbreeding coefficient (*F*_ROH_) of each goat group.

Breeds	Number	Obs_Hetezygosity	Exp_Hetezygosity	*F*_ROH_
ABS	10	0.144751	0.217544	0.517070
ALS	9	0.149858	0.223363	0.522920
CDM	27	0.262376	0.273373	0.076754
ELS	10	0.173097	0.241556	0.427173
GS	6	0.273914	0.238914	0.036232
HG	5	0.238966	0.221172	0.159380
LG	5	0.205116	0.188409	0.278300
LL	15	0.22665	0.232717	0.202993
LN	13	0.133443	0.218359	0.541562
TB	6	0.233774	0.23865	0.178202
TS	15	0.230768	0.245215	0.192126
UC	10	0.266199	0.258638	0.063418
YN	20	0.25967	0.251541	0.086361
YS	6	0.259122	0.232894	0.088293

## Data Availability

All the data were downloaded from NCBI. All scripts used for this work were performed using open-source software tools and are available from the corresponding authors upon request.

## Supplementary Materials

The following supporting information can be downloaded at: https://www.mdpi.com/article/10.3390/genes16030292/s1, Table S1: Whole sequencing data information of goats in this study. Table S2: Six climate variables of each population. Table S3: The top 5% of candidate genes under selection in cashmere goats by *F_ST_*. Table S4: The top 5% of candidate genes under selection in cashmere goats by θπ. Table S5: The top 5% of candidate genes under selection in cashmere goats by XP-EHH. Table S6: Overlapping selection candidate genes for cashmere goats by *F_ST_*, *θ*π, and XP-EHH. Table S7: GO analysis for the regional candidate genes selected in cashmere goat. Table S8: KEGG analysis for the regional candidate genes selected in cashmere goat. Table S9: Overlapping selection candidate genes for cashmere goats by *F_ST_* and BAYPASS. Table S10: GO & KEGG analysis for the regional candidate genes with Genome-wide scans for local adaptation significant association.
